# De novo inference of stratification and local admixture in sequencing studies

**DOI:** 10.1186/1471-2105-14-S5-S17

**Published:** 2013-04-10

**Authors:** Yu Zhang

**Affiliations:** 1 Department of Statistics, The Pennsylvania State University 326 Thomas Building, University Park, PA 16802, USA

## Abstract

Analysis of population structures and genome local ancestry has become increasingly important in population and disease genetics. With the advance of next generation sequencing technologies, complete genetic variants in individuals' genomes are quickly generated, providing unprecedented opportunities for learning population evolution histories and identifying local genetic signatures at the SNP resolution. The successes of those studies critically rely on accurate and powerful computational tools that can fully utilize the sequencing information. Although many algorithms have been developed for population structure inference and admixture mapping, many of them only work for independent SNPs in genotype or haplotype format, and require a large panel of reference individuals. In this paper, we propose a novel probabilistic method for detecting population structure and local admixture. The method takes input of sequencing data, genotype data and haplotype data. The method characterizes the dependence of genetic variants via haplotype segmentation, such that all variants detected in a sequencing study can be fully utilized for inference. The method further utilizes a infinite-state Bayesian Markov model to perform *de novo *stratification and admixture inference. Using simulated datasets from HapMapII and 1000Genomes, we show that our method performs superior than several existing algorithms, particularly when limited or no reference individuals are available. Our method is applicable to not only human studies but also studies of other species of interests, for which little reference information is available.

Software Availability: http://stat.psu.edu/~yuzhang/software/dbm.tar

## Introduction

Recent advance in high-throughput sequencing technologies [1-3] has enabled genome-wide identification of genetic variants at the individual level. Particularly, single nucleosome polymorphism (SNP) is the most common and the easiest genetic information detected by sequencing. SNPs not only contain rich information about the evolution of individuals, but also can be used as markers to pinpoint phenotype-causative loci in phenotype-ascertained samples. Sequencing technologies can detect all mutations genome-wide. The complete genetic landscape thus provides us with unprecedented opportunities to learn the evolution history of individuals and identify functional regions with phenotypic consequences at the SNP resolution. The complexity and the scale of sequencing data, however, impose new computational and statistical challenges that require development of new methodologies.

In this paper, we introduce a new method for identifying population stratification (or population structure) and local admixture for sequencing studies. Sensitive population structure detection and high-resolution inference of local ancestry have wide applications in disease genetics [4-8]. Population stratification refers to non-random mating between groups of individuals (often due to physical separation), such that there is a systematic difference in the SNP allele frequencies between groups. One can detect population stratification by clustering analysis, where individuals within clusters have similar allele frequencies across SNPs, and individuals between groups have different allele frequencies. STRUCTURE [9] is based on this idea, yet STRUCTURE and many other approaches [10-13] require independent SNPs for *de novo *structure detection, i.e., identifying unknown numbers of populations in a sample. To analyze all SNPs from sequencing studies, methods that can handle linkage disequilibrium (LD) among SNPs are needed.

Population admixture is a reverse process of stratification, where two or more previously separated populations begin interbreeding. The genomes of admixed individuals therefore contain genetic information from multiple lineages as a mosaic combination. If the history of admixing is relatively recent, we can trace back the ancestry of each genomic region in admixed individuals by comparing the region to that of non-admixed individuals with known ancestry. Many methods [14-22] have been developed for local ancestry inference in admixed populations, but again many of them require independent SNPs and thus cannot be applied to sequencing data. In addition, ancestry information is hard to obtain except for human studies, such that existing methods cannot be used.

We introduce a new method called DBM-Admix (Dynamic Bayesian Markov model for Admixture mapping) for detecting population stratification and mapping local admixtures in sequencing studies. Compared to existing methods, DBM-Admix has several advantages. 1) The method can perform *de novo *inference of stratification and admixture, i.e., without requiring reference ancestry information. 2) The method can accommodate switching errors in haplotype phasing. Several existing methods infer admixture in each haplotype separately, assuming that the input haplotypes have no switch errors, which is unrealistic and can loose power. 3) As opposed to modeling individuals separately and/or utilizing sliding windows, our method makes inference of all individuals simultaneously, and uses Markov chains to infer local admixture at the SNP resolution.

DBM-Admix is the first algorithm for *de novo *mapping of local admixtures using all SNPs without pre-screening independent and/or ancestry informative (AIM) SNPs. The main difficulty of *de novo *mapping lies in that, without knowing the dependence structure of SNPs, there are no standard criteria to determine the number of populations and admixtures. Our method tackles this problem by first learning the SNP dependence structure using an infinite-state hidden Markov model. It then uses the learned SNP dependence and combines all individuals to detect unknown population structures and local admixtures via a Bayesian probabilistic model. An advantage of Bayesian approaches is that model uncertainties and regularization are naturally taken into account by Bayesian priors. As a result, DBM-Admix works well even if little and possibly unreliable reference information is available.

Our method dynamically partitions individuals' genomes into states (the number of states is unknown if ancestral information is unavailable). Our approach has two layers of hierarchies: 1) one layer of hidden Markov model (HMM) for characterizing SNP dependence in haplotypes, where sequencing data are converted into haplotype segments; and then 2) another layer of HMM for population admixture, where the haplotype segments are clustered into populations. Transitions between haplotype segments and populations are allowed to represent haplotype recombination and population admixture, respectively. For computational efficiency and also for practical interests, we separate the two layers of HMMs into two programs. We first infer haplotype structures (haplotypes and their segmentations) from sequencing data using our previously developed method DBM-Hap [23], and then we run DBM-Admix to further identify population stratification and local admixture.

For *de novo *mapping of stratification, we compare DBM-Admix to fineSTRUCTURE [24], which is currently the only other program that can do *de novo *stratification detection on dependent SNPs. For local admixture mapping, we compare DBM-Admix to three benchmark methods: HAPMIX [18], PCADMIX [21], and LAMP-LD [22]. These methods have very different mechanisms for admixture inference and can all handle LD between SNPs (PCADMIX automatically filters SNPs in strong LD and thus serves as a benchmark of independent SNP method). None of the above methods directly take sequencing data as inputs, but they can be applied after converting sequencing reads into genotypes/haplotypes.

## Results

### Simulated datasets

We downloaded the phased haplotypes of individuals from the HapMap project [25] and the 1000 Genomes project [26], respectively. Using these haplotypes, we simulated new individuals by randomly recombining haplotypes within and across populations (while the latter is admixing) according to pre-specified proportions. The frequency of recombing haplotypes within a population is 1 per 200 kb. The probability of admixing across populations at each SNP *j *is 1-exp(-*λd_j_*), where *λ*denotes the number of generations of admixture, and *d_j _*denotes probability of crossover between SNPs (*j*-1) and *j *in one generation. For HapMapII samples, *d_j _*is given by the HapMap genetic map in centimorgan (1% probability of recombination per generation). For 1000Genome samples, we used linear interpolation to calculate *d_j _*from the HapMap genetic map. We further simulated stratified individuals as a special case when *λ *= 0. The benchmarking programs fineSTRUCTURE, HAPMIX, LAMPLD and PCADMIX require input of either haplotypes (PCADMIX) or genotypes (HAPMIX and LAMPLD), and if reference individuals are used, they all require haplotype format of reference individuals. Although genotypes and haplotypes are already given in the simulated sample, they serve as the "truth" in this study and thus cannot be directly used as the input to each program. Instead, we simulated sequencing data (with a Poisson distribution) at 8× coverage from the true genotypes and re-called genotypes and re-phased haplotypes from the simulated sequencing data by DBM-Hap [23]. We then removed "non-polymorphic" SNPs from the reconstructed data and input the inferred genotypes and haplotypes to each program. The simulated sequencing coverage is large enough so that the genotyping error rate is <1% and the haplotype phasing error rate is <5%, representing realistic errors encountered in practice. Some previous methods did not do this additional step and thus their results are over-optimistic.

### *De novo *inference of population stratification

**Figure 1:**
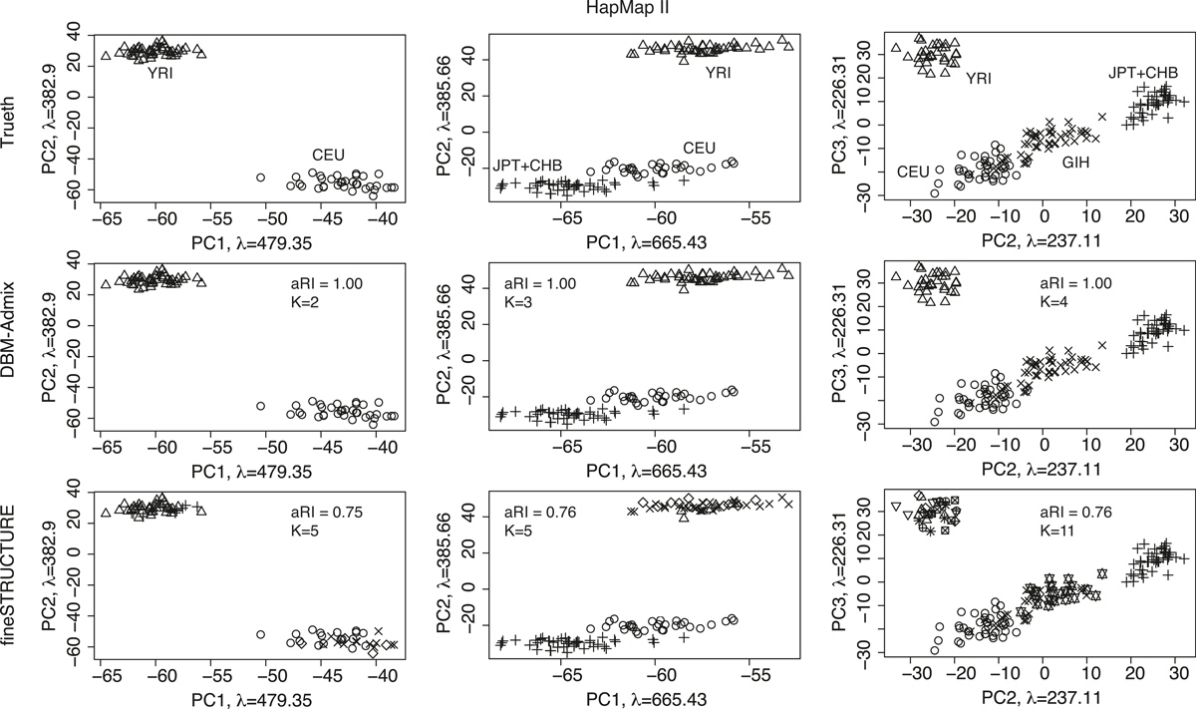
**Comparison of *de novo *structure inference in HapMapII data**. Each plot shows individuals (haplotypes) in different origins (shown in symbols) projected onto the principal components (*λ *denotes eigen values). Top: true origins. Middle: origins inferred by DBM-Admix. Bottom: origins inferred by fineSTRUCTURE. Columns from left to right correspond to datasets containing 2, 3 and 4 populations, respectively. aRI: adjusted rand index. K: estimated number of populations.

We first evaluated DBM-Admix for *de novo *inference of population stratification. Because of the strong LD among SNPs, the only method we can compare to is fineSTRUCTURE [24], while all other *de novo *stratification algorithms work for independent SNPs only, and cannot identify the correct number of populations on dependent SNPs. We simulated datasets containing *K *= 2, 3 and 4 populations from the HapMapII data and the 1000Genomes data, respectively. Particularly, for the HapMapII data, the populations are (CEU, YRI), (CEU, YRI, JPT+CHB), and (CEU, YRI, JPT+CHB, GIH), respectively. For the 1000Genomes data, the populations are (CEU, YRI), (CEU, YRI, JPT), and (CEU, YRI, JPT, MXL), respectively. In each dataset, we simulated 20 diploid individuals per population, and each individual contained 10,000 HapMapII SNPs and 30,000 1000Genomes SNPs, respectively, which covered ~8 Mb region randomly chosen in the genome. We ran DBM-Admix and fineSTRUCTURE on these datasets in default settings. Figure 1 shows the results of the HapMapII datasets, where DBM-Admix identified all individuals' origins perfectly and also inferred the correct number of populations in each dataset. In contrast, fineSTRUCTURE consistently over-estimated the true number of populations in all datasets, and the detected population structures were inaccurate. We measured the accuracy of the inferred population structures by the adjusted rand index (aRI) [27], by which aRI = 1 means 100% correct and aRI = 0 means random guessing. The adjusted rand index can measure consistency between two clustering results even if their numbers of clusters are different. It is seen that fineSTRUCTURE split the individuals within the same populations into subpopulations. This appeared to be positively correlated with the total number of individuals in each dataset (40, 60, and 80 for *K *= 2, 3, and 4, respectively), and was not due to real subpopulations in the data, because we randomly recombined haplotypes to generate new individuals in each population. The over-estimation of fineSTRUCTURE is likely due to its *ad hoc *use of the tuning parameter *c*, which failed to correctly adjust for the effective number of independent SNPs.

**Figure 2:**
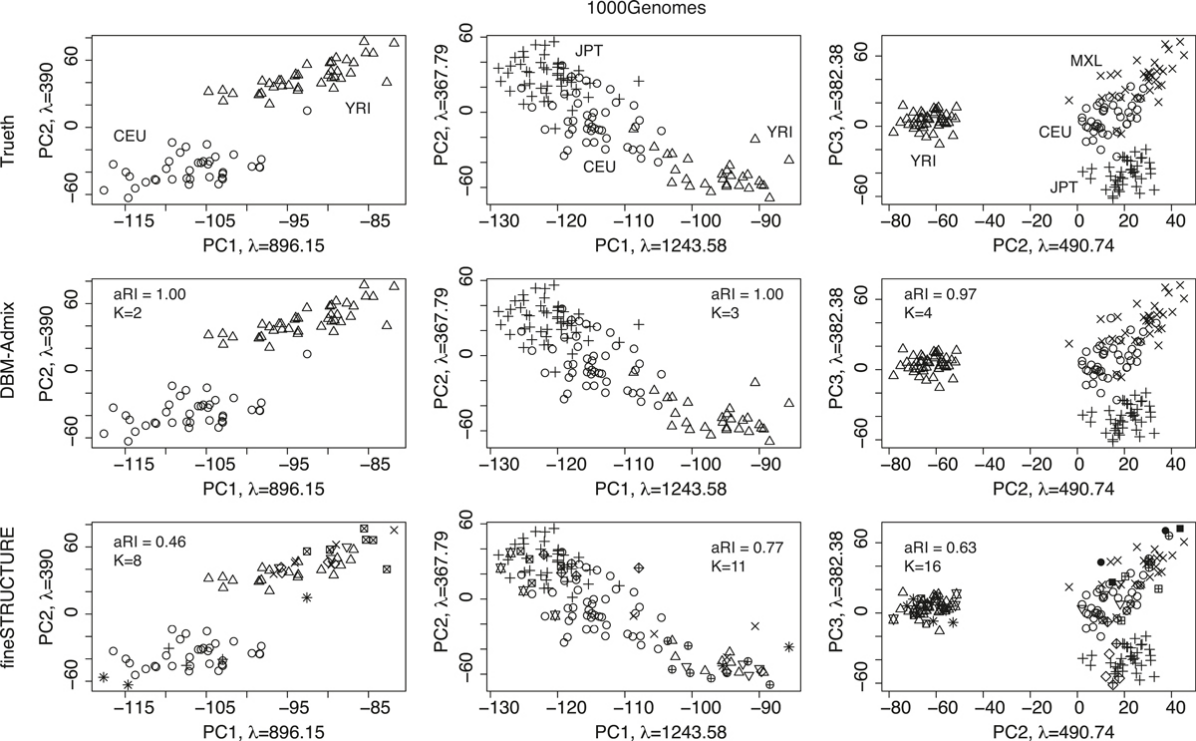
**Comparison of *de novo *structure inference in 1000Genome data**. Each plot shows individuals (haplotypes) in different origins (shown in symbols) projected onto the principal components (*λ *denotes eigen values). Top: true origins. Middle: origins inferred by DBM-Admix. Bottom: origins inferred by fineSTRUCTURE. Columns from left to right correspond to datasets containing 2, 3 and 4 populations, respectively. aRI: adjusted rand index. K: estimated number of populations.

Figure 2 shows the results of the 1000Genomes datasets. Again, DBM-Admix performed very well with only one mistake at *K *= 4. In contrast, the results of fineSTRUCTURE were much worse than those obtained in the HapMapII data, both in the adjusted rand index and in the estimated number of populations. It is seen from the principal component projection that the individuals in HapMapII were more separated than individuals in 1000Genomes. The 1000Genomes data contained many SNPs not in HapMapII, the haplotype configurations of which were relatively similar across populations. As a result, it is harder to analyze the 1000Genomes data, for which fineSTRUCTURE performed unsatisfactorily.

### Local admixture mapping with references

**Figure 3:**
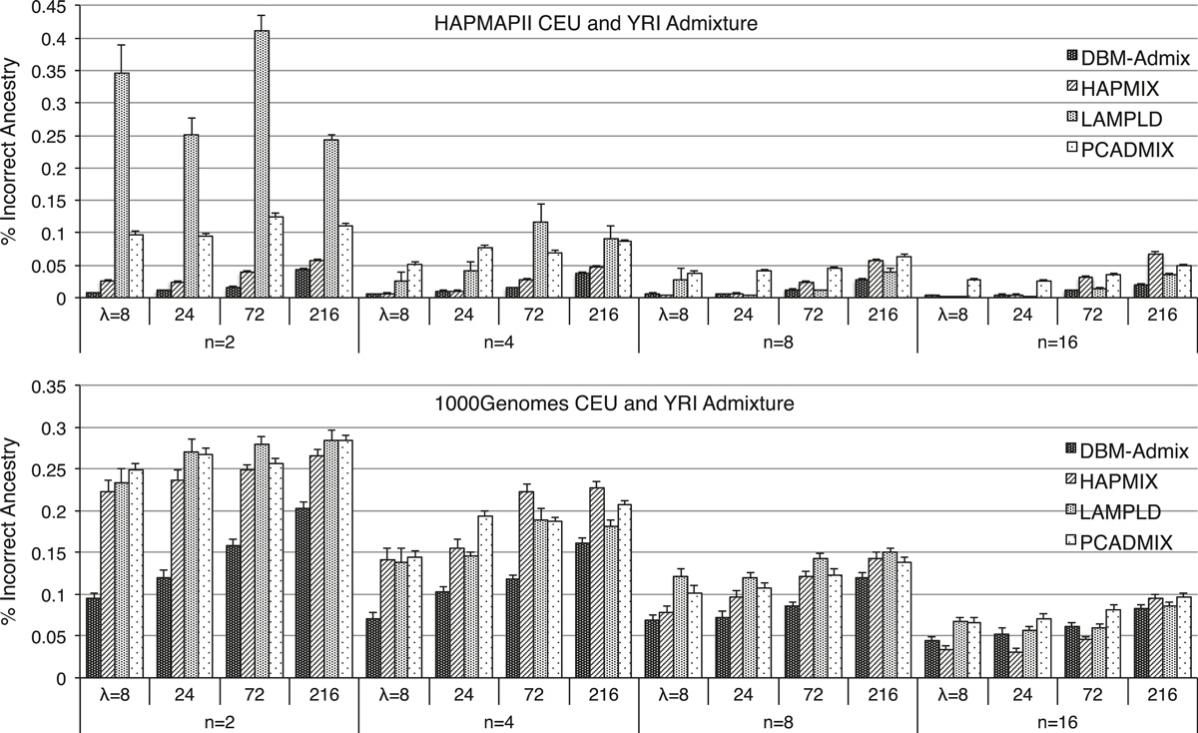
**Percentage of incorrect local ancestries with standard errors inferred by DBM-Admix, HAPMIX, LAMPLD and PCADMIX in individuals admixed with equal proportions of CEU and YRI origins from HapMapII and 1000Genomes, respectively**. Individuals are admixed by *λ *= 8,24,72,216 generations and inferred using *n *= 2,4,8,16 references with known origins from each population.

We next evaluated DBM-Admix for local admixture inference using ancestral references. We first simulated 2-way admixture datasets containing 20 individuals with equal proportions of CEU and YRI origins at 40,000 HapMapII and 120,000 1000Genome SNPs, respectively. The number of SNPs was chosen such that each dataset covered ~30 Mb region in the genome. Figure 3 shows the percentage of incorrect local ancestry inferred by DBM-Admix, HAPMIX, LAMPLD, and PCADMIX in samples admixed by *λ *= 8, 24, 72, 216 generations, using n = 2, 4, 8, 16 ancestral references per population, respectively. We did not use the adjusted rand index here, because each admixed individual may carry haplotypes from multiple origins. The percentage of incorrect local ancestry is calculated at each SNP separately and then averaged over all SNPs. We observed that DBM-Admix performed consistently and substantially better than the other methods when only *n *= 2 reference individuals per ancestral population were available. At *n *= 4, DBM-Admix still performed better than the other methods in all cases. At *n *= 8, DBM-Admix performed the second best after LAMPLD for the HapMapII data, but performed the best for the 1000Genomes data. At *n *= 16, DBM-Admix still performed the 2^nd ^best in all cases. In addition, the errors for all methods increased as *λ *increased, i.e., more ancient admixtures are harder to identify. The 1000Genomes data were again harder to analyze and had much higher error rates than the HapMapII data..

**Figure 4:**
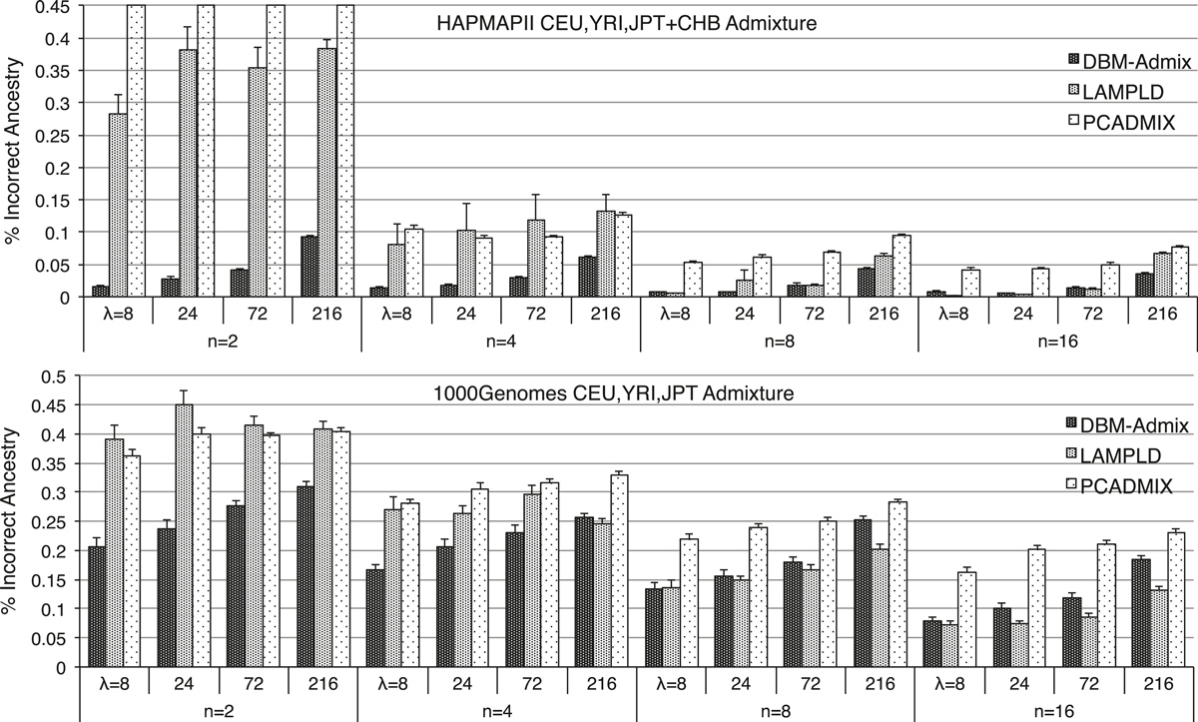
**Percentage of incorrect local ancestries with standard errors inferred by DBM-Admix, LAMPLD and PCADMIX in individuals admixed with equal proportions of CEU, YRI and JPT(+CHB) origins from HapMapII and 1000Genomes, respectively**. Individuals are admixed by *λ *= 8,24,72,216 generations and inferred using *n *= 2,4,8,16 references with known origins from each population.

We next simulated datasets of 3-way admixtures containing 20 individuals admixed with equal proportions of CEU, YRI and JPT (+CHB) origins at 40,000 HapMapII SNPs and 120,000 1000Genome SNPs, respectively. We dropped HAPMIX from this study as it only works for 2 populations. Figure 4 shows the proportion of incorrect local ancestry inferred by the three programs. Similar to the results of 2-way admixture, and more evidently, DBM-Admix performed substantially better than the other methods when the number of reference individuals was small (at *n *= 2 and 4). With more references used, LAMPLD began to perform similar (*n *= 8) or better (*n *= 16 1000Genomes data) than our method, while PCADMIX performed the worst in most scenarios (at *n *= 2, PCADMIX failed to produce any results for the HapMapII data due to singularity problems). This suggests that selecting independent and/or AIM SNPs is not desirable and is less powerful than using all SNPs.

**Figure 5:**
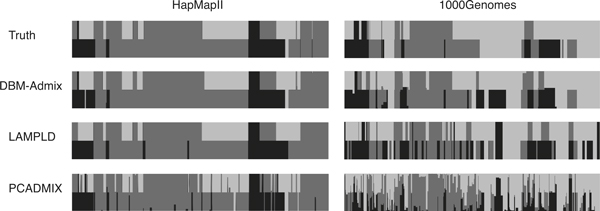
**Examples of 3-way admixtures inferred by DBM-Admix, LAMPLD and PCADMIX using 4 ancestral references per population**. The three populations are CEU (black), YRI (dark grey), and JPT(+CHB) (light grey) from HapMapII and 1000Genomes, respectively.

We show in Figure 5 two examples of 3-way admixture inference results using *n *= 4 references per ancestral population, for HapMapII and 1000Genomes, respectively. The results inferred by DBM-Admix, LAMPLD and PCADMIX for only one individual are shown. We observed that the results for HapMapII were much cleaner than the results for 1000Genomes. Although the error rates for the 1000Genomes data (right panel in Figure 5) were large (>20%), our method still produced good agreement between the inferred and the true local ancestries in most regions. Comparing the results of the 3 methods, DBM-Admix produced the most accurate and the cleanest inference, whereas PCADMIX produced the noisiest results with spurious spikes, which is probably due to its inefficient selection of AIMs from limited references. Finally, although we only showed the results of equal proportions of admixtures in this study, we have further tested all methods on datasets with unequal proportions of admixtures (e.g., 80% CEU and 20% YRI, data not shown), where we obtained almost the same results and conclusions.

### *De novo *local admixture mapping

Our method can in principle identify unknown numbers of populations admixed in a sample using a dynamic Bayesian Markov process. We have already shown its performance in *de novo *identification of population structures. For admixture mapping, however, *de novo *inference is much more difficult, because not only the number of populations is unknown, the locations and the frequency of local admixtures are also unknown. We tested DBM-Admix without using references in two ways: 1) "0 ref": no reference data but specify the population number; and 2) "*de novo*": no reference data and no population number. We tested the method on the datasets simulated in the 2-way and 3-way admixture studies with 2 references, and we call the previous results of DBM-Admix "2 ref" as a benchmark. Without reference information, it is not guaranteed for the method to yield the correct population labels and/or identify the correct number of populations at all SNPs. When comparing the results, therefore, we performed label mapping at each SNP. In particular, we ran DBM-Admix on the admixed individuals along with 2 reference individuals per population, without telling the program the origins of the references. We then mapped the inferred population labels to the true labels of the references to maximize their correlation. Finally, we computed the percentage of incorrect local ancestries using the mapped labels on the admixed individuals.

Table 1 shows the result of DBM-Admix for *de novo *admixture mapping. For the HapMapII datasets, our method performed similarly among the three input types, with "2 ref" slightly better than "0 ref", and "0 ref" slightly better than "*de novo*", which were consistent with the amount of information we provided to the program. For the 1000Genomes datasets, we observed similar results but with larger error rates. The results of "0 ref" and "*de novo*" were almost identical in the 1000Genomes data, suggesting that using references are more critical when analyzing individuals admixed between similar populations or when the data are noisier.

**Table 1:** Percentage of incorrect local ancestry inferred by DBM-Admix with and without using reference.

	2-way admix								3-way admix							
	**HapMapII**				**1000Genomes**				**HapMapII**				**1000Genomes**			

*λ*	8	24	72	216	8	24	72	216	8	24	72	216	8	24	72	216

2 ref	0.7	1.1	1.6	4.3	9.5	12.1	15.8	20.3	1.7	2.6	4.1	8.7	20.8	23.8	27.7	30.9

0 ref	1.0	1.7	2.9	5.5	14.8	17.9	22.1	25.6	3.0	4.2	7.7	15.1	26.0	31.6	36.5	35.2

*denovo*	2.0	2.4	3.5	8.4	15.2	20.6	21.6	25.6	2.9	5.4	9.0	18.1	25.1	31.8	38.3	35.3

"*λ*": # of admix generations; "2 ref": 2 references per population; "0 ref": no reference but specify # of populations; "*denovo*": no reference and no # of populations.

**Figure 6:**
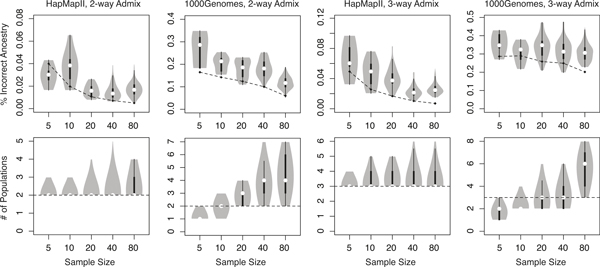
***de novo *local admixture inference by DBM-Admix at different sample sizes**. Top: violin plots of proportion of incorrect local ancestries; dashed lines show the "2 ref" results. Bottom: inferred SNP-wise number of populations; dashed lines show the true numbers of populations.

We further evaluated the performance of *de novo *admixture mapping of DBM-Admix with respect to sample size. We simulated datasets of 5, 10, 20, 40 and 80 individuals (at *λ *= 24 and two references per population for label mapping) with 2-way and 3-way admixtures from HapMapII and 1000Genomes data, respectively, following the same simulation procedures as described above. As shown in Figure 6 (top), the error rates of local admixture decreased as sample size increased, because our method combined multiple individuals for joint admixture inference. The error rates of *de novo *inference were greater than the error rates of "2 ref" (using 2 references per population) in most cases, but the differences were not substantial. We further show in Figure 6 (bottom) the number of admixing populations per SNP inferred by DBM-Admix. It is very challenging to identify the correct number of admixing populations (dash lines) at all SNPs, particularly for the 1000Genomes data, but our method performed satisfactorily. These results suggested that *de novo *local admixture mapping is indeed feasible in certain scenarios.

## Discussion

We have presented a novel method DBM-Admix for detecting population stratification and admixture requiring little information about ancestral populations. One motivation of this work is that in many sequencing studies, particularly exploratory studies, it is very expensive to obtain samples with known ancestry from a species of interest. Particularly ancestral populations may have been extinct in many species. In such cases, existing methods will perform poorly or fail to produce results. DBM-Admix can take input of sequencing data, genotype data, and haplotype data. The method incorporates LD information through haplotype segmentation, which is internally inferred by a method called DBM-Hap (paper submitted), such that all SNPs are jointly utilized without requiring pre-screening of independent and/or AIM SNPs.

Compared to existing methods, DBM-Admix is advantageous in that it infers structures of all individuals simultaneously, such that information is borrowed across individuals to help detecting subtle structures. DBM-Admix is also robust to switch errors in haplotype phasing. Switching pieces of haplotypes within an individual can hamper the power of admixture mapping. This is seen from the fact that more frequent admixture is harder to infer, whereas switching errors due to computational phasing algorithms can artificially create extra "admixtures". DBM-Admix is built on a Bayesian framework so that inference uncertainty is accounted for in the model. Particularly, when there are few reference individuals, the uncertainty (or reliability) of the reference information, such as the ancestral allele frequencies, can be automatically taken into account by the model. As a result, DBM-Admix avoids over fitting the data. Finally, DBM-Admix learns the dependence structure of SNPs in a sample and utilizes the dependence to perform *de novo *detection of stratification and local admixture. The idea is to use a Bayesian Markov process to find a proper number of states to fit the data. With SNP dependence captured by haplotype segments, DBM-Admix is able to estimate the number of populations stratified or admixed in a sample. Using simulated datasets from two very different reference panels, HapMapII and 1000Genomes, we demonstrated the superior performance of our method compared to existing approaches, with and without using ancestral references.

In term of computing speed, DBM-Admix runs linearly with respect to the sample size and the number of SNPs if the number of populations is fixed. For *de novo *inference, DBM-Admix runs proportional to the square of the number of populations inferred by the program. For example, DBM-Admix took ~5 minutes to infer each of the 1000Genomes results in Figure 3 and 4, which contained 20 individuals and 120,000 SNPs per individual. For the *de novo *mapping results in Figure 6, without knowing the true number of populations, our method took ~10 minutes. About the same amount of time is further required to run DBM-HAP to obtain haplotype segmentation, but this time can be reduced if haplotypes are given.

The current DBM-Admix model can be improved in several ways. One drawback in the current model is that the method makes a *bona fide *use of haplotype segmentation. If the segmentation is inaccurate, the power of DBM-Admix will suffer. A simple solution is to run the method multiple times independently and then summarize results from all runs. Alternatively, we may merge DBM-Hap and DBM-Admix together into a joint hierarchical model and simultaneously infer haplotypes and population structures. We avoided this approach not only because of its obvious computational burdens, but also because haplotype inference by itself is of interest in many studies (e.g., disease association studies). The users may also want to use haplotypes obtained by other means to infer population structure and admixture. Another weakness of the current model is that, although haplotype segmentation captures SNP dependence, haplotype segments are not equally similar or dissimilar in allele composition. A population is more likely to carry similar haplotype segments, yet the current model does not take this information into account. A possible extension of DBM-Admix is thus to introduce a hierarchical relationship between haplotype segments, such that a population carrying one haplotype segment is more likely to carry another haplotype segment with similar genetic contents. This idea has been previously used in haplotype inference [28], which is straightforward to implement.

## Methods

### Haplotype segmentation

We first use DBM-Hap [23] to infer haplotype structures from sequencing data. Note that haplotype structures are not equivalent to haplotypes. Haplotypes are just allele compositions across SNPs, whereas haplotype structures further include allele dependence information and the locations of recombination events. We infer haplotype structures by DBM-Hap [23], which is briefly described below.

The input of DBM-Hap is sequencing read counts of two alternative alleles per SNP per individual, denoted by *D*={*d_ij_*}, for *i *= 1,...,*N *individuals and *j *= 1,...,*L *SNPs, where *d_ij _*= (*A_ij_*,*a_ij_*) denotes the read counts for alleles *A *and *a*, respectively. We assume that all individuals are unrelated. We introduce a *2NL *binary matrix *H *= {h_ij1_, h_ij2_}, for *i *= 1,...,*N*, *j *= 1,...,*L*, denoting the haplotypes of *N *individuals at *L *SNPs, where (*h_i•1_*, *h_i•2_*) denotes the haplotype pair for individual *i*, and *h_ijl _*= 0,1 indicates the absence and presence of minor alleles, respectively. To learn haplotype structures, we introduce a latent variable *S *= {*s*_ij1_, *s*_ij2_}, for *i *= 1,...,*N*, *j *= 1,...,*L*, denoting the haplotype states for *N *individuals at *L *SNPs. *S *represents *2N *Markov chains, where (*s_i•1_*, *s_i•2_*) represents a pair of Markov chains for individual *i*, and *s_ijl _*takes any positive integer values, i.e., infinite number of states, denoting the index of haplotype state of the *l*^th ^haplotype at SNP *j *in individual *i*. At each SNP, we assume that haplotypes in the same state have a common allele frequency. Individuals' haplotype states at nearby SNPs tend to be identical due to its Markov nature. As a result, similar haplotypes will be assigned into the same states. Our intuition is to capture the "ancestral" haplotypes by *S*, the diversity of which is much lower than that of haplotypes (*H*) in the current sample. To identify recombination events, we further introduce an indicator variable *Φ *= {*φ*_ij1_,*φ*_ij2_} denoting the transition between states in the *2N *Markov chains across *L *SNPs. The joint model of DBM-Hap is therefore written as Pr(*D*,*H*,*S*,*Φ *) = Pr(*D*|*H*)Pr(*H*|*S*,*Φ *)Pr(*S*,*Φ *). In this model, Pr(*D*|*H*) denotes the probability of read counts given haplotypes, which we model by Poisson distributions. Pr(*H*|*S,Φ*) denotes the emission probability of alleles given states, which we model by independent Bernoulli events at each SNP. Pr(*S*,*Φ *) denotes the Markov chains of haplotype states, which we model by a dynamic infinite state Bayesian Markov process. The output of DBM-Hap includes the posterior inference of haplotype states *S*, recombination events *Φ *, and recombination probabilities {*r_j_*}*_j = _*_1*,...,L *_at each SNP. These yield haplotype segmentation at the individual level and are used as the input to DBM-Admix.

### DBM-Admix model

A haplotype segment contains an interval of SNPs that belong to the same haplotype state in *S*, and the segment is bounded by two recombination events specified by *Φ *. Conceptually, each haplotype segment represents a piece of ancestral haplotypes, within which alleles are inherited together to the current population. The haplotype segments therefore capture the allele dependence across SNPs. We directly use the segmentation results from DBM-Hap to infer population structure and local admixture. The idea is to introduce another layer of HMMs representing population ancestries, where individuals from the same population, in a region, have the same distribution of haplotype segments.

Let *X *= {*X_ik1_*,*X_ik2_*}, for *i *= 1,...*N *and *k *= 1,2,..., denote the haplotype segments in *N *individuals, with *X_ikl _*= {*s_ijl_*}, for *j *= (1≤)*a_ikl_*,*a_ikl_*+1,...,*b_ikl_*-1,*b_ikl _*(≤*L*) and *l *= 1 or 2, denoting the *k*^th ^haplotype segment of the *l*^th ^haplotype in individual *i*. The interval [*a_ikl_*, *b_ikl_*] is given by *Φ *from DBM-Hap and is treated as fixed. Also, haplotype segments are consecutive, i.e., *b_i(k-1)l _*+1 = *a_ikl_*. We next introduce *2N *Markov chains to model population ancestry. Let *Q *= {*q_ij1_*,*q_ij2_*} denote the population states, for *i *= 1,...,*N*, *j *= 1,...,*L*, where *q_ijl _*takes any positive integer values denoting indices of population origins. Again, we allow infinite number of populations. Further let *I *= {*I_ij1_*,*I_ij2_*}denote the indicators of population admix events in individual *i *at SNP *j*. We write the joint probability function of (*X*, *Q*, *I*) in the form of

(1) Pr(X,Q,I)=Pr(X|Q,I)Pr(Q,I)

where Pr(*X*|*Q*,*I*) denotes the emission probability of haplotype segments given population states and admix events, and Pr(*Q*,*I*) denotes the HMM distribution of population origins.

To model Pr(*X*|*Q,I*), we first identify double haplotype recombination sites in all individuals. The double recombination sites are the SNPs at which both haplotypes in an individual recombine. These are the sites of potential haplotype switch errors. For convenience, we further denote the two ending SNPs (SNP 1 and SNP L) as double recombination sites. Let {*E_im_*} denote the collection of haplotype segments lie between the *m^th ^*and the (*m+*1)^th ^double recombination sites in individual *i*. Let *δ_im _*denote an indicator of whether or not a haplotype switch error occurs at the double recombination site *m *in individual *i*, we write

(2) $$\text{Pr}\left(X|Q,I\right)\propto \prod_{i=1}^{N}\left[\sum_{\left\{\delta_{im}\right\}}\prod_{m}\varepsilon^{\left|\delta_{im}-\delta_{i\left(m-1\right)}\right|}\prod_{k\in E_{im}}\prod_{l=1}^{2}\prod_{j=a_{ikl}}^{b_{ikl}}\text{Pr}{\left(s_{ijl}|q_{ijl}\right)}^{\left(1-\delta_{im}\right)w_{ijl}}\text{Pr}{\left(s_{ij\left(3-l\right)}|q_{ijl}\right)}^{\delta_{im}w_{ij\left(3-l\right)}}\right]$$

In formula (2), Pr(*s_ijl_*|*q_ijl_*) denotes how frequent a haplotype state *s_ijl _*occurs in population *q_ijl_*, which is a parameter estimated iteratively as described in the *Model Fitting *section. Pr(*s_ij_*_(*3-l*)_|*q_ijl_*) denotes the similar parameter but with the haplotype pair switched (when *δ*_im _= 1, population *q_ijl _*on the *l*^th ^strand emits haplotype state *s_ij_*_(*3-l*) _on the (3-*l*)^th ^strand, for *l *= 1 or 2). We assign a small weight *w_ijl _*to the power of Pr(*s_ijl_*|*q_ijl_*) and Pr(*s_ij_*_(*3-l*)_|*q_ijl_*) to adjust for the fact that haplotype states within a segment are redundant information. By default, $w_{ijl}=\left(1+\sum_{j^{\prime }=a_{ikl}+1}^{b_{ikl}}r_{j^{\prime }}\right)/\left(b_{ikl}-a_{ikl}+1\right)$, for *a_ikl_*≤*j*≤*b_ikl_*, where *r_j' _*is the haplotype recombination probability at SNP *j' *provided by DBM-HAP. The numerator of the weight equals to the expected number of haplotype recombination events within segment [*a_ikl_*, *b_ikl_*], and the denominator equals to the total number of SNPs within the segment. As a result, $\prod_{j=a_{ikl}}^{b_{ikl}}\text{Pr}{\left(s_{ijl}|q_{ijl}\right)}^{w_{ijl}}$in formula (2) equals to the geometric mean of {Pr(*s_ijl_*|*q_ijl_*)} over all SNPs in the segment to the power of the expected number of haplotype recombination events. Another possible choice of weight is to let *w_ijl _= *1 at *j *= *a_ikl_*, i.e., the first SNP in each segment, and *w_ijl _= r_j _*otherwise, which produces similar results. In formula (2), we also sum over all possible haplotype switch errors at all double recombination sites, and we let the switch error probability *ε *= 0.5.

We next model the HMM distribution Pr(*Q*,*I*) of population ancestries. To detect an unknown number of stratification and admixtures, we use an infinite state Markov model that automatically determines the number of states at each SNP. Let {*v_q_*} denote an infinite dimensional vector of probabilities that sum to 1, and is used as the "ancestral" distribution of population states. We model the prior distribution of {*v_q_*} by a stick-breaking process [29]. Let {*V_q_*} denote an infinite set of independent *Beta *random variables, with *V_q_*~*Beta*(1,1), we express *v_q _*= *V_q_*∏*_t < q_*(1-*V_t_*). Using this prior, DBM-Admix essentially allows an infinite number of populations to be fitted to the data. Simultaneously, *v_q _*is regularized, because it tends to 0 with probability approaching to 1 as *q *increases to infinity, and hence avoids over-fitting the data. Let {*γ*_j_} denote the population admix probability between SNPs *j*-1 and *j*. We write

$$\text{Pr}\left(Q,I|\left\{\nu_{q}\right\}\right)=\prod_{i=1}^{N}\prod_{l=1}^{2}\text{Pr}\left(Q_{i∙l},I_{i∙l}|\left\{\nu_{q}\right\}\right)=\prod_{i=1}^{N}\prod_{l=1}^{2}\overset{L}{\underset{j=2}{\prod }}v_{q_{i1l}}\overset{L}{\underset{j=2}{\prod }}\left[{\left(1-\gamma_{j}\right)}^{1-I_{ijl}}{\gamma_{j}}^{I_{ijl}}v_{q_{ijl}}^{I_{ijl}}\right]$$

subject to the constraint that, if *q_i(j-1)l_*≠*q_ijl _*(i.e., an admix event between SNPs (*j-*1) and *j*), the admix indicator *I_ijl _*must be 1 (and vise versa, if *I_ijl _*= 1, then *q_i(j-1)l _*= *q_ijl_*), otherwise the probability equals to 0. Note that our model has heterogeneous transition probabilities across SNPs. Let ***v ***= {*v_q_*} denote an infinite-dim column vector of population distribution, **1 **denote an infinite-dim column vector of 1s, our transition probability matrix at SNP *j *is given by diag(1-*γ*_j_, ∞)+*γ*_j_**1*v***'.

Without knowing {*γ*_j_}, we assign a Dirichlet prior *Dir*(*αr_j_*,*1*-*αr_j_*) to {*γ*_j_}, where *r_j _*is the haplotype recombination probability output by DBM-Hap, and *α *denotes a small constant (by default 0.2). Let *ξ_j _*= ∑*_i_*∑*_l_I_ijl _*denote the total number of admix events at SNP *j *in all Markov chains, we integrate out {*γ*_j_} and obtain the marginalized probability function

(3) $$\text{Pr}\left(Q,I|\left\{\nu_{q}\right\}\right)=\left(\overset{N}{\underset{i=1}{\prod }}\overset{2}{\underset{l=1}{\prod }}v_{q_{i1l}}\overset{L}{\underset{j=2}{\prod }}v_{q_{ijl}}^{I_{ijl}}\right)\overset{L}{\underset{j=2}{\prod }}\frac{\Gamma \left(\xi_{j}+\alpha r_{j}\right)\Gamma \left(2N-\xi_{j}+1-\alpha r_{j}\right)\Gamma \left(1\right)}{\Gamma \left(2N+1\right)\Gamma \left(\alpha r_{j}\right)\Gamma \left(1-\alpha r_{j}\right)}$$

Derivation of formula (3) is almost identical to the derivation of formula (4) in [23], from which more details can be found.

Putting formulas (2) and (3) together, along with the prior distribution of {*v_q_*}, we obtain the full probability function in the form of Pr(*X*|*Q,I*)Pr(*Q,I*|{*v_s_*})Pr({*v_q_*}). The unknown parameters in our model include population origins *Q*, population admix events *I*, distribution of population origin {*v_q_*}, and haplotype segment emission probability Pr(*s_ijl_*|*q_ijl_*). All these parameters are inferred iteratively as described below.

### Model fitting

Starting from a random initialization of parameters *Q*, *I*, {*v_q_*}, we first update the population-specific haplotype state distribution Pr(*s_ijl_*|*q_ijl_*) = (*y_ijl _*+ 1)/(*n_ijl_*+*K_j_*), where *y_ijl _*denotes the number occurrences of haplotype *s_ijl _*and population *q_ijl_*, *n_ijl _*denotes the total number of haplotypes in population *q_ijl_*, and *K_j _*denotes the number of distinct haplotype states at SNP *j*.

Given Pr(*s_ijl_*|*q_ijl_*), we next update *Q *and *I *from the full model, iteratively for one individual at a time conditioning on the parameters of the other individuals. For each individual *i*, we update {*q_i.1_*,*q_i.2_*} and {*I_i.1_*,*I_i.2_*} using a forward-summation and backward-sampling (or maximization) algorithm. In the forward-summation step, we calculate the marginal probability of a specific configuration of population origins ending at SNP *j *of individual *i*, where the population origins and the admix events at SNPs 1,...,*j*-1 are marginalized out. This is done recursively at SNPs *j *= 1,...,*L *in ascending order. To handle infinite number of states, we collapse all origins with indices >q* into a "super" state, where q* denotes the maximum index in *Q *in the current iteration. The number of distinct population origins therefore becomes finite in computation. In the backward-sampling (or maximization) step, we use the calculated marginal probabilities to update {*q_i•1_*,*q_i•2_*} and {*I_ij1_*,*I_ij2_*} at SNPs *j *= *L*,...,1 sequentially in descending order. In particular, we first determine {*q_iL1_*,*q_iL2_*} by sampling from (or maximizing) the marginal probability at SNP *L*, and we let *I_iL1 _*= *I_iL2 _*= 0. If a "super" state is chosen, indicating a new population, we further determine the label of the new population from the prior distribution. Next, at each SNP *j < L *in descending order, we first determine the admix events {*I_ij1_*,*I_ij2_*} according to the marginal probability at SNP *j *and conditioning on the origins {*q_i_*_(*j+1*)_*_1_*,*q_i_*_(*j+1*)_*_2_*} obtained at SNP (*j*+1). We then determine {*q_ij1_*,*q_ij2_*} based on {*I_ij1_*,*I_ij2_*} and {*q_i_*_(*j+1*)_*_1_*,*q_i_*_(*j+1*)__*2*_}. If admix does not occur at SNP *j *in the *l^th ^*haplotype (*I_ijl _*= 0), then *q_ijl _*= *q_i(j+1)l_*. Otherwise, a new population is sampled in the same way as described for SNP *L*. In practice, either backward sampling or maximization works well, but sampling can help alleviating local mode problems and thus is used by default. We further restrict that admix can only occur at the boundaries of haplotype segments.

Finally, we update the distribution of population origins {*v_q_*}. Let {*c_q_*} denote the total occurrence of population *q *at either admix sites (*I_ijl _*= 1) or the start of Markov chains. We sample *V_q _*from *V_q_*~*Beta*(*c_q_*+1, *∑_t>q_c_t_*+2), which is the posterior distribution of *V_q_*. We then calculate *v_q _*by *v_q _*= *V_q_*∏*_t < q_*(1-*V_t_*). Note that we only need to calculate {*v_q_*} for a finite number of origins up to q*, because we collapse all unoccupied origins with indices >q* into a "super" state, the posterior probability of which is 1-∑_q_≤_q* _v_q_.

We repeat the above model fitting procedures iteratively and we allow a few iterations of burn-in before we collect posterior samples of parameters of interest. To avoid local mode problems, we also randomly split population origins a few times during burn-in, such that the algorithm has a chance to detect more subtle population structures. Finally, we infer population structure and admixture by maximum a posteriori from the posterior samples, at each SNP separately. If reference individuals with known ancestries are available, say, from *C *ancestries (*C *= 2,3,...), then DBM-Admix reduces to a heterogeneous (with respect to transition probabilities) Markov model with fixed number of states, and it fits both the sample and the reference individuals together.

## Competing interests

The author declare that he has no competing interests.

## Authors' contributions

YZ designed and carried out the entire study and wrote the manuscript.

## Declarations

Publication of this article was supported by NIH R01HG004718 and NIH 1UL1RR033184.

This article has been published as part of *BMC Bioinformatics *Volume 14 Supplement 5, 2013: Proceedings of the Third Annual RECOMB Satellite Workshop on Massively Parallel Sequencing (RECOMB-seq 2013). The full contents of the supplement are available online at http://www.biomedcentral.com/bmcbioinformatics/supplements/14/S5.
